# The Herb Pair *Radix Rehmanniae* and *Cornus Officinalis* Attenuated Testicular Damage in Mice With Diabetes Mellitus Through Butyric Acid/Glucagon-Like Peptide-1/Glucagon-Like Peptide-1 Receptor Pathway Mediated by Gut Microbiota

**DOI:** 10.3389/fmicb.2022.831881

**Published:** 2022-02-22

**Authors:** Yuping Chen, Siyuan Song, Anmei Shu, Liping Liu, Jinjin Jiang, Ming Jiang, Qin Wu, Huiqin Xu, Jihu Sun

**Affiliations:** ^1^Department of Basic Medical Science, Jiangsu Vocational College of Medicine, Yancheng, China; ^2^Department of Science and Technology, Jiangsu Vocational College of Medicine, Yancheng, China; ^3^School of Pharmacy, Jiangsu Vocational College of Medicine, Yancheng, China; ^4^School of Medical Technology, Jiangsu Vocational College of Medicine, Yancheng, China; ^5^School of Medicine, Jiangsu Vocational College of Medicine, Yancheng, China; ^6^School of Pharmacy, Nanjing University of Chinese Medicine, Nanjing, China

**Keywords:** *Radix Rehmanniae*, *Cornus Officinalis*, butyric acid, diabetes-associated reproductive damage, gut microbiota

## Abstract

Growing body of research indicates that Traditional Chinese Medicine (TCM) interact with gut microbiota (GM) after oral administration. *Radix Rehmanniae* and *Cornus Officinalis* (RR-CO), a well-known TCM pair, is often used to treat diabetes mellitus (DM) and its complications. The current study aimed to explore the protective effects of RR-CO on DM induced testicular damage by modulating GM. The RR-CO treatments significantly reduced hyperglycemia, ameliorated testicular ultrastructural damage and inflammation in DM model to varying degrees. Additionally, 16S-ribosomal DNA (rDNA) sequencing results showed that RR-CO treatment increased the amount of butyric acid-producing GM, such as *Clostridiaceae_1* family, and decreased the abundance of *Catabacter*, *Marvinbryantia*, and *Helicobacter* genera. RR-CO fecal bacteria transplantation (RC-FMT) increased the abundance of *Clostridiaceae_1* in the Model FMT (M-FMT) group and ameliorated testicular damage. Furthermore, treatment with RR-CO increased the fecal butyric acid level, serum Glucagon-like peptide-1 (GLP-1) level, and testicular GLP-1 receptor (GLP-1R) expression compared to those in DM mice. Finally, intraperitoneal administration of sodium butyrate (SB) significantly improved the pathological damage to the testis and reduced inflammation in the DM group. These data demonstrated a protective effect of RR-CO on DM-induced testicular damage by modulation of GM, which may be mediated by the butyric acid/GLP/GLP-1R pathway.

## Graphical Abstract

A diagram illustrating the alleviating effect of the herb pair RR-CO on testicular damage in diabetes mellitus by modulating the butyrate/GLP-1/GLP-1R pathway mediated by gut microbiota.

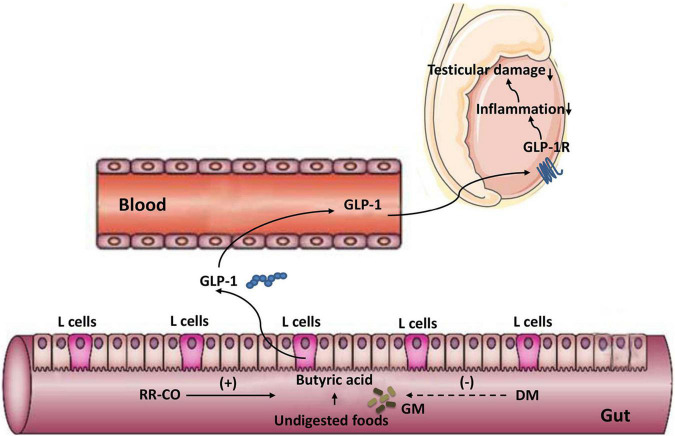

## Introduction

Diabetes mellitus (DM) is a metabolic disease characterized by chronic hyperglycemia caused by deficiency of insulin secretion or impairment of insulin action. The most common complications of diabetes are diabetic cardiomyopathy, nephropathy, and retinopathy. Recently, researches have focused on the influence of DM on reproductive function. Sexual dysfunction is common in men with diabetes, and about 15% of men with primary infertility have undiagnosed prediabetes (Boeri et al., 2020). The main clinical manifestations of male reproductive damage caused by diabetes are abnormal hormone levels, decreased libido, erectile dysfunction, decreased spermatogenesis and fertility (Maresch et al., 2018). In the pathogenesis of male infertility with diabetes, hyperglycemia may lead to the release of large amounts of superoxides and other free radicals into the cytoplasm to increase mitochondrial glucose oxidation, and then damage the tissue structures of the testis, epididymis, and blood-testis barrier, accompanied by sperm malformation (Tian et al., 2020). In addition, diabetic testicular vascular disease is also an important cause of diabetic testicular damage. Long et al. (2018) found that the testicular blood flow velocity in diabetic rats was significantly lower than that in the control group, which may be linked to the damage of testicular blood vessel microcirculation caused by hyperglycemia. Other mechanisms include disturbances in the hypothalamic–pituitary–gonadal axis, affecting the hormone levels of testosterone (T), follicle-stimulating hormone (FSH), and luteinizing hormone (LH) (Shi et al., 2017).

Recent findings showed an association between the testicular function and regulation of gut microbiota (GM) *via* host metabolites. Zhang et al. (2021b) suggested that the modulation of GM altered bile acid levels and further affected vitamin A absorption, resulting in damaged spermatogenesis in the metabolic syndrome model. There is a close association between high-fat diet induced microbiota dysbiosis, defect in spermatogenesis with elevated endotoxins, dysregulation of testicular gene expression, and epididymal inflammation (Ding et al., 2020). El-Baz et al. (2021) spotted the beneficial effect of Lactobacillus in combination with montelukast and metformin in reducing the testicular damages of chronic diabetic rat model through modulation of GM. Our previous studies have also revealed a strong link between diabetic testicular damage and dysbiosis of GM (Liu et al., 2021; Zhu et al., 2021). Nevertheless, the contribution of GM to reproductive damage in the diabetic environment is not intensively documented.

Advances have been made in the development of treatments for DM-induced male reproductive dysfunction but most drugs are characterized by poor long-term efficacy and associated secondary side effects. Traditional Chinese Medicine (TCM) has the advantages of better efficacy and lighter side effects. *Rehmanniae Radix*, the root of *Rehmannia glutinosa* Libosch. (RR; family, Scrophulariaceae), decreases body heat, cools the blood, nourishes Yin, and produces fluid. It is often used to treat chronic metabolic diseases including DM and its complications (Kim et al., 2017; Qi et al., 2019). *Corni Fructus* (*Cornus officinalis* Sieb. et Zucc.; CO; family, Cornaceae) nourishes the liver and kidney and has a long history of treating diabetes. It is often prescribed in TCM prescriptions, such as Zan Yu Dan and Liuwei Dihuang Pill, treating spermatorrhea and reproductive disorders, respectively (Park et al., 2015; Bi et al., 2019). Our previous studies have shown that *Radix Rehmanniae* and *Cornus Officinalis* (RR-CO) attenuated DM-induced testicular damage by regulating glucose metabolism, but the underlying mechanism needs to be further clarified.

Recently, with the increase in GM research, the interaction between TCM, chronic metabolic diseases, and GM has attracted much attention. Human GM is a complex micro-ecosystem, while TCM is characterized by being “multi-component and multi-targeted.” Several recent studies have shown that TCM helps maintain GM homeostasis and *vice versa*, and GM may regulate the pharmacological effects of TCM (Feng et al., 2019). Accordingly, the involvement of GM in the prevention and treatment of type 2 diabetes mellitus (T2DM) has also been worked upon. Chen et al. (2018) reported that the Huang-Lian-Jie-Du-Decoction ameliorated hyperglycemia and restored the dysregulated microbiota structure and function, mainly by increasing short chain fatty acid (SCFA)-producing bacteria and reducing conditioned pathogenic bacteria in T2DM rats. Extracts of CO have been demonstrated to exhibit curative hypoglycemic effects and selectively modulate GM in T2DM mice (Niu et al., 2020). Zhang et al. (2021c) implied that GM and their metabolites might play a vital role in the progression of chronic kidney disease, which was reversed by the treatment with *Rehmannia Radix Preparata* and CO herb pair. However, hitherto, the research on the gut-testis axis in metabolic diseases is still limited. Herein, we investigated whether the herb pair RR-CO could attenuate DM-induced testicular damage by modulating GM and affecting metabolism. These findings represent an addition to the functions of the gut-testis axis *via* the host metabolome.

## Materials and Methods

### Reagents and Antibodies

*Radix Rehmanniae* and *Cornus Officinalis* raw herbs were purchased from Haichang Chinese Medicine (Jiangsu, China, Batch No. 190101) and identified by a professor from Nanjing University of Chinese Medicine; the voucher numbers of CO (No. 20170505) and RR (No. 20181020) were deposited. The herbs were cut into small pieces, soaked in 10 volumes of distilled water for 30 min, and boiled twice. To prepare RR-CO, the two original herbs were mixed at a ratio of 2:1, which is a common dose in clinical practice. The final doses of RR, CO, and RR-CO were 200, 100, and 300 mg/mL, respectively. Antibodies against IL-6 (Batch No. sc-57315) and TNF-α (Batch No. sc-52B83) were obtained from Santa Cruz Biotechnology (United States). Antibodies against NF-κB (Batch No. 10745-1-AP) were purchased from Wuhan Proteintech Group (China). Antibodies against Glucagon-like peptide-1 receptor (GLP-1R) (Batch No. bs-1559R) were purchased from Beijing Bioss Biotechnics Co., Ltd. (China).

### Animal Model Establishment

**FIGURE 1:**
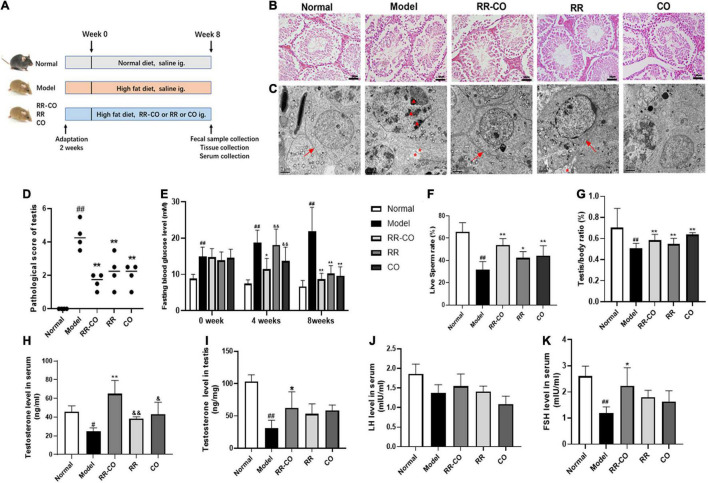
The effects of RR-CO on testicular morphological injury, fasting blood glucose, sperm motility, and testosterone level in DM mice. **(A)** Flow chart of animal study design. **(B)** H&E staining of mice testicular tissues. Scale bar, 50 μm. **(C)** Transmission electron microscopic observation of testicular ultrastructure of mice (*blacktriangle*, nuclear chromatin; *, cytoplasmic vacuolation; →, tight junctions). Scale bar, 2 μm. **(D)** A chart showing testicular lesion scores vs. model group. **(E)** FBG levels over the 8-week experiment. **(F)** Live sperm rate. **(G)** Testis/body weight ratio. **(H)** Serum testosterone level. **(I)** Testicular testosterone level. **(J)** Serum LH level. **(K)** Serum FSH level. Data are expressed as mean ± SD. ^#^*P* < 0.05; ^##^*P* < 0.01 vs. normal group. *P < 0.05; **P < 0.01 vs. model group. ^&^*P* < 0.05; ^&&^*P* < 0.01 vs. RR-CO group.

Ten-week-old male C57BL/6J and KK-Ay mice (license number: 2014-0004) were purchased from Beijing Huafukang Biotechnology Co., Ltd. The mice were bred adaptively for 2 weeks, and the temperature and humidity conditions maintained in the animal house were 25 ± 5°C and 55 ± 5%, respectively. All the animal experiments performed were approved by the Animal Ethics Committee of the Nanjing University of Traditional Chinese Medicine (code: 201903A019). C57BL/6J mice were fed a normal diet, while KK-Ay mice were fed a high-fat diet (458 kcal/100 g, containing 10% fat). Then, the KK-Ay mice were randomly divided into RR-CO, RR, and CO groups. The three groups were given RR-CO (3,000 mg/kg/d), RR (2,000 mg/kg/d), and CO (1,000 mg/kg/d), respectively, for 8 weeks. The KK-Ay mice in the model group were administrated saline. Seven C57BL/6J mice that were fed with a normal diet were also given saline. At the end of the experiment, the mice were sacrificed using the cervical dislocation technique, and blood was drawn from the orbit and serum was extracted. Testes were resected and weighed to calculate the ratio of the bilateral testis to body weight. The flow chart of the animal study is shown in Figure 1A.

### Determination of Live Sperm Rate, Testosterone, Follicle-Stimulating Hormone and Luteinizing Hormone Level

The tail of the epididymis was homogenized in PBS and incubated at 37°C for 5 min to release the sperms. The suspension was then mixed with 1% trypan blue of the same volume and observed using an optical microscope. Live sperm eliminated the dye, while the dead sperm accumulated it and presented a blue head. The serum and testicular testosterone level was detected using enzyme-linked immunosorbent assay (ELISA) kits (Anhui Joyee Biotechnics Co., Ltd., China), the FSH and LH were measured using ELISA kit from Shanghai Jianglai Biotechnics Co., Ltd., China.

### Histological Morphology and Transmission Electron Microscopy and Immunofluorescence Assay

The testicular tissue was fixed with 4% paraformaldehyde, embedded in paraffin, sliced, dewaxed with xylene and ethanol, then stained with hematoxylin and eosin (H&E), and sealed with neutral gum. Use a microscope to photograph randomly selected areas. The semi-quantitative scoring of the H&E image was conducted according to the grading arrangement and structure of spermatogenic cells, interstitial cell proliferation, interstitial vascular dilatation, congestion, bleeding, inflammatory cell infiltration, etc. For transmission electron microscopy (TEM) assay, the testicular tissue was fixed with 2.5% glutaraldehyde solution at 4°C for 24 h, followed by gradient dehydration and imaging of the seminiferous tubule at random fields. Immunofluorescence staining was performed as follows: paraffin sections of mouse testes were dewaxed, rehydrated in xylene and ethanol, and then incubated for 20 min at 37°C in proteinase K working solution, after PBS rinsing, the cells were incubated with primary antibody against GLP-1R (dilution 1:200) for 2 h at room temperature, followed by secondary antibody with FITC fluorescence, nuclei were stained with DAPI for 5 min and a Zeiss LSM 900 confocal microscope Images were taken and immunofluorescence intensity was quantified using ImageJ software.

### Measurement of Cecal Short Chain Fatty Acids

The major SCFAs in mice feces of all groups, such as acetic acid, propionic acid, butyric acid, and valeric acid, were detected using gas chromatography–mass spectrometry (GC-MS, Thermo Scientific TSQ 8000, United States). About 50 mg of feces of each mouse were collected, and 1,000 μL 0.005 mol/L NaOH aqueous solution was added to each sample separately. After vortexing for 10 min, the feces were centrifuged at 4°C at 10,000 rpm for 15 min. About 500 μL of the supernatant was taken and added to a 10-mL glass centrifuge tube. Then, 300 μL pure water, 500 μL propanol pyridine solution, and 100 μL n-propyl chloroformate were added to the above extract. The mixture was vortexed for 10 s and subjected to ultrasound sonication for 1 min. After the derivatization, n-hexane was used to extract the derivatized substances. First, 300 μL n-hexane was added to the reaction system, and the sample was centrifuged at 3,000 rpm for 5 min. About 200 μL of the supernatant was taken in an injection bottle, and 200 μL n-hexane was added to the reaction system again, followed by vortexing for 1 min and centrifugation for 5 min at 3,000 rpm. Another 200 μL of the supernatant was taken into the injection bottle and 60 μL of the supernatant was used for GC-MS analysis.

### Gut Microbiome 16S Ribosomal DNA V3–V4 Region Illumina PE250 Sequencing

According to the characteristics of the amplified 16S ribosomal DNA (rDNA) region using the Illumina Miseq technology sequencing platform, a small fragment library was constructed using paired-end sequencing. Complete standard information analysis and advanced information analysis were performed to analyze the microbial diversity in the sample. Genomic DNA of the gut microbiome (GM) was extracted from fecal samples and the bacterial V3–V4 region of the 16S rDNA gene from each sample was amplified. Sequencing was performed using an Illumina Miseq PE250 platform (Shanghai Bioprofile Technology Co., Ltd.). Raw reads were stored in the NCBI database.

### Western Blotting

Testis and colon tissue was lysed with RIPA buffer (Solarbio Life Sciences, Beijing, China) to produce lysate. Protein sample (15–30 μg per sample) was separated using 10% SDS-PAGE polyacrylamide gel, and then transferred to the PVDF membrane. Then the membrane is blocked with 5% skimmed milk for 2 h, incubated with the primary antibody, corresponding horseradish peroxidase-conjugated secondary antibodies were then followed. At the end, protein bands were detected using a chemiluminescence kit (Millipore, United States) with ECL chemiluminescence system (Tanon-5200Multi, Shanghai, China). Data was qualified by ImageJ software. The experiments were repeated three times.

### Fecal Bacteria Transplantation Experiment

A total of 100 mg of fresh stool samples from the Model and RC groups were collected immediately and resuspended in 1 mL of saline, vortexed for 5 min, and filtered using a sterile nylon net. Then, 20% glycerin was added to the bacterial suspension and the suspension was stored at −80°C. Twelve C57BL/6J recipient mice were fed a high-fat diet (FB-D12492, 60% fat, Wuxi Fanbo Biotechnology Co., Ltd) for 1 month and injected with 60 mg/kg Streptozotocin twice a week to establish the type 2 diabetes model. Furthermore, six C57BL/6J mice on a normal diet without STZ injection were used as normal control. The recipient mice were then given water containing antibiotics (1 g/L ampicillin, 1 g/L metronidazole, 0.5 g/L vancomycin, and 0.5 g/L neomycin) for 2 weeks and then gavaged with 200 μl of fecal sample supernatant every day for another 3 weeks. The flow chart of the FMT animal study was shown in Figure 5A.

**FIGURE 2:**
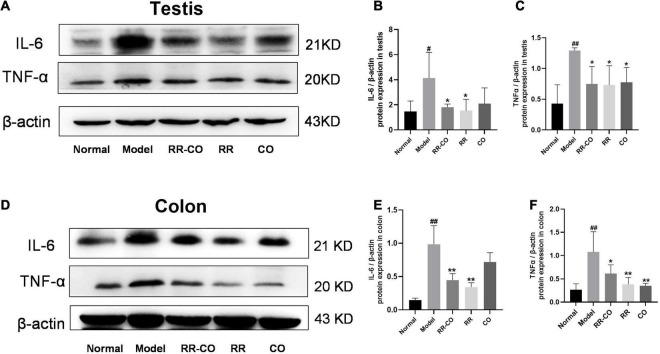
The effects of RR-CO on inflammation in the testes and colon of DM mice. **(A–C)** The expression of IL-6 and TNF-α in testicular tissue of DM mice. **(D–F)** The expression of IL-6 and TNF-α in the colon of mice. Data are expressed as mean ± SD. ^#^*P* < 0.05; ^##^*P* < 0.01 vs. normal group. *P < 0.05; **P < 0.01 vs. model group; *n* = 3.

**FIGURE 3:**
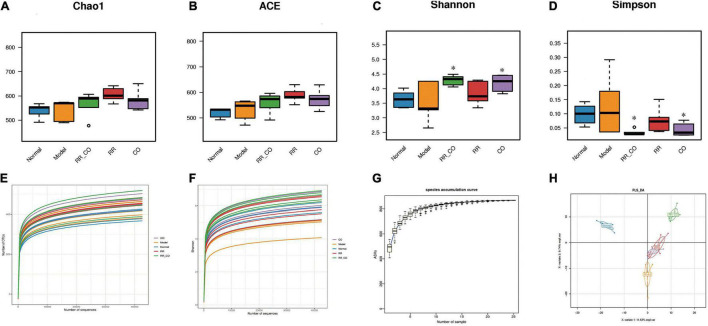
The effects of RR-CO on GM diversity in DM mice. **(A,B)** The Chao1 and ACE indices were calculated to determine the total number of species in the five groups. **(C,D)** The Shannon and Simpson’s indices were calculated to identify the diversity of GM. **(E)** Rarefaction analysis of 16S rDNA V3–V4 region *via* Illumina PE250 sequencing in GM. **(F)** Shannon curve of V3–V4 region *via* Illumina PE250 sequencing in GM. **(G)** Species accumulation curves. **(H)** The differences in community composition identified using PLS-DA. Data are expressed as mean ± SD. *P < 0.05 vs. model group; *n* = 5.

**FIGURE 4:**
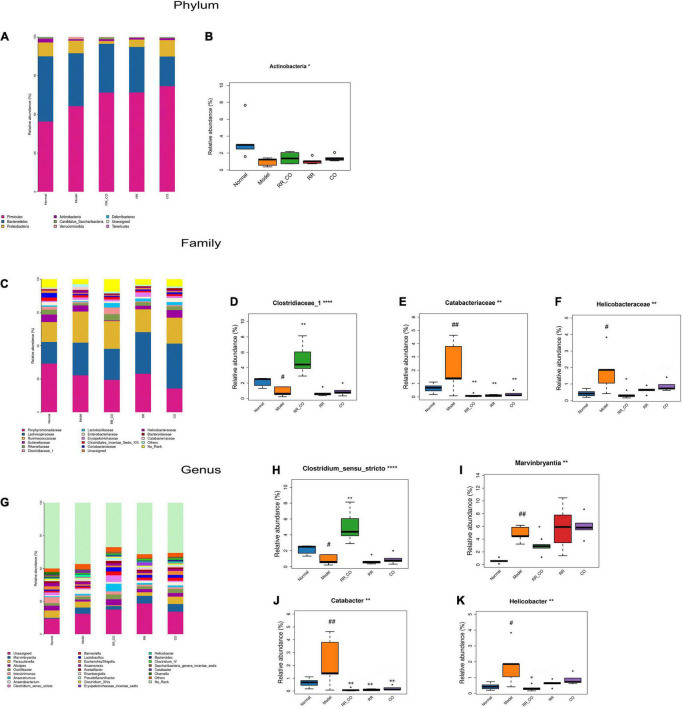
The effects of RR-CO on the abundance of GM in DM mice. **(A,B)** The relative abundance of the main phyla of GM in different groups. **(C–F)** The relative abundance of the main families of GM in different groups. **(G–K)** The relative abundance of the main genus of GM in different groups. Data are expressed as mean ± SD. ^#^*P* < 0.05; ^##^*P* < 0.01 vs. normal group. *P < 0.05; **P < 0.01 vs. model group; *n* = 5.

**FIGURE 5:**
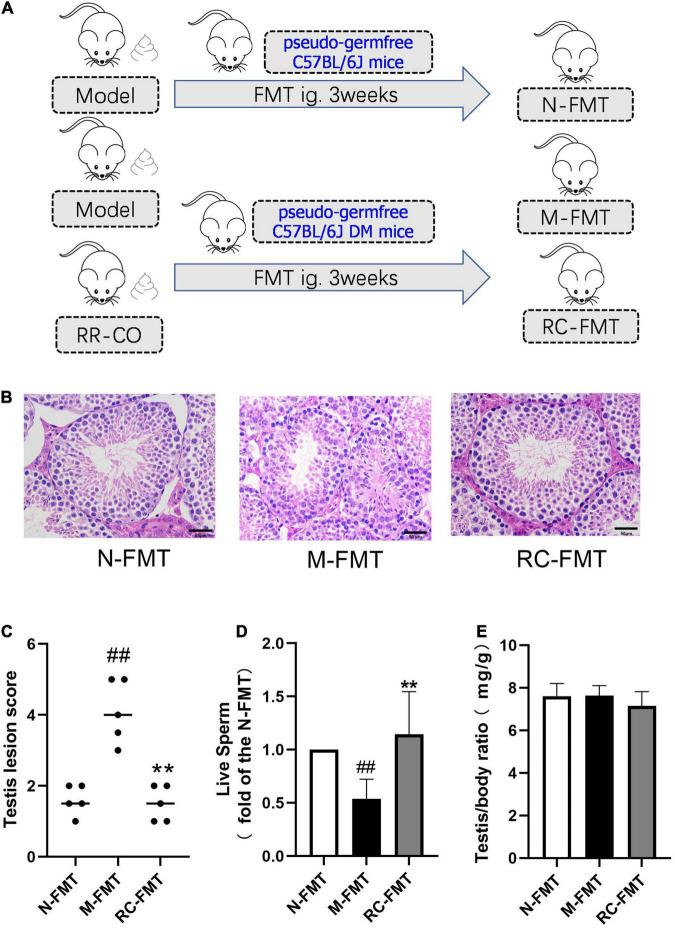
The effects of FMT on DM-induced testicular damage. **(A)** Flow chart of FMT animal study. **(B)** H&E staining of mice testicular tissues. Scale bar, 50 μm. **(C)** A chart showing testicular lesion scores. **(D)** A chart showing the live sperm rate (a fold of the N-FMT). **(E)** Testis/body weight ratio. Data are expressed as mean ± SD. ^##^*P* < 0.01 vs. N-FMT; **P < 0.01 vs. M-FMT; *n* = 5.

### Statistical Analysis

The data were expressed as mean ± standard deviation (SD) and analyzed by one-way ANOVA with Spss19.0 software, and then the differences between groups were analyzed using LSD *t*-test, the correlations were statistically evaluated using Pearson correlation tests with Spss19.0 software, *P* < 0.05 showed statistical difference.

## Results

### *Radix Rehmanniae* and *Cornus Officinalis* Treatment Improved Diabetes Mellitus-Induced Ultrastructural Damage to the Testis, Decrease in Testosterone Levels, and Degradation in Sperm Parameters

Hematoxylin and eosin results demonstrated that all stages of spermatogenic cells were abundant and orderly with filamentous sperm cells in the lumen in the normal group, while in the model group, the spermatogenic layer was destroyed and the cells were disorganized. Also, the mature sperm cells in the lumen decreased in the model group, suggesting that the spermatogenic function of diabetic animals was reduced (Figures 1B,D). Compared to the normal group, the TEM results showed an uneven distribution of nuclear chromatin, the chromatin particles concentrated to the heterochromatin region, the tight junctions between cells were damaged, and the vacuolization of cytoplasm increased in the model group. The study of the testicular ultrastructure in the RR-CO group showed that the tight junctions between the cells were clear, vacuolization was ameliorated, and the nuclear chromatin was well-distributed (Figure 1C). The RR-CO, RR, and CO groups showed reduced fasting blood glucose (FBG) level and an increase in the live sperm rate, serum and testicular testosterone level, and testis/body weight ratio to varying degrees compared to the model group (Figures 1E–I). The serum FSH level in the model group was also dramatically decreased compared with the normal group, while RR-CO administration can increase the level. Nonetheless, serum LH levels did not show significant differences between the groups (Figures 1J,K).

### *Radix Rehmanniae* and *Cornus Officinalis* Treatment Alleviated the Increased Inflammatory Responses in the Colon and Testes of Diabetes Mellitus Mice

Western blotting was performed to evaluate the inflammatory status of the testicular and colon tissues. The results revealed that, compared to the normal group, the expression of pro-inflammatory factors IL-6 and TNF-α significantly increased in the model group, while treatments with RR-CO, RR, and CO down-regulated their expression to varying degrees (Figures 2A–F).

### The Effects of *Radix Rehmanniae* and *Cornus Officinalis* Treatment on the Diversity of Gut Microbiota in Diabetes Mellitus Mice

Chao1 and ACE indices were used to estimate the total number of microbial species in the sample, showing no significant differences across the groups (Figures 3A,B). Shannon and Simpson’s indices represent the diversity of the bacterial community. The Shannon index in the RR-CO and CO group was significantly higher than that in the model group, whereas the Simpson’s index was lower than that in the model group (*P* < 0.05) (Figures 3C,D). The rarefaction curve already reached stable values, suggesting that the amount of sequencing data is reasonable (Figure 3E), while the Shannon curve indicated that the sequencing data reflected majority of GM information in the sample (Figure 3F). The species accumulation curves also reached a plateau, indicating that the species in this environment will not increase significantly with the increase in sample size, implying that the sampling was sufficient (Figure 3G).

Partial Least Squares Discriminant Analysis (PLS-DA) was used to study the differences in sample community composition. PLS-DA results indicated that there were significant structural differences in the GM across the normal, model, and RR-CO groups (Figure 3H).

### *Radix Rehmanniae* and *Cornus Officinalis* Treatment Restored the Altered Gut Microbiota Abundance in Diabetes Mellitus Mice

To further explore the differences in the structure of GM across groups, the bacteria from each sample were analyzed at the phylum, family, and genus levels. The histogram shows the overall community structure of GM and the top nine relatively abundant phyla of GM in different groups (Figure 4A). The relative abundance of *Actinobacteria* was lower in the model group compared to the normal group, while RR-CO groups showed an increase in the abundance compared to the model group (Figure 4B). At the family level, 17 predominant families in the five groups are shown in Figure 4C. The relative abundance of *Clostridiaceae_1* was dramatically lower in the model group compared to the normal group, whereas RR-CO increased the abundance of *Clostridiaceae_1* significantly (Figure 4D). Meanwhile, the relative abundance of certain pathogenic microorganisms, such as *Catabacteriaceae* and *Helicobacteraceae*, increased significantly in the model group and decreased in the normal, RR-CO, RR, and CO groups to varying degrees (Figures 4E,F). At the genus level, 26 intestinal bacteria were found to be significantly differentially abundant across the five groups, and the abundance level of four genera reversed after the treatments (Figure 4G). *Catabacter*, *Marvinbryantia*, and *Helicobacter*, which increased in the model group, were down-regulated with RR-CO, RR, or CO treatment to a varying degree. On the contrary, the decreased numbers of *Clostridium_sensu_stricto* in the model group were restored in the RR-CO group. However, RR and CO alone did not significantly upregulate the abundance of *Clostridium_sensu_stricto* (Figures 4H–K).

### Fecal Microbiota Transplantation Remodeled Gut Microbiota and Alleviated Diabetes Mellitus-Induced Testicular Damage

To further verify the role of GM dysbiosis in diabetes-induced reproductive damage, we conducted FMT experiments, transplanting feces of DM (model group) and RR-CO-treated DM mice into the recipient pseudo-germfree C57BL/6J mice with or without diabetic symptoms (Figure 5A). Feces of the model group (M-FMT) resulted in the aggravation in the testicular damage, manifested as an abnormal structure of the spermatogenic cells and a reduction in the number of mature spermatogenic cells in the lumen. Also, the live sperm rate in the M-FMT group showed a significant reduction compared to the Normal FMT (N-FMT) group. Conversely, RR-CO FMT (RC-FMT) group mice demonstrated remarkable attenuation in the testicular pathological damage and an up-regulation in the live sperm rate (Figures 5B–D). However, there was no significant difference in testis/body weight ratio across the three groups (Figure 5E).

**FIGURE 6:**
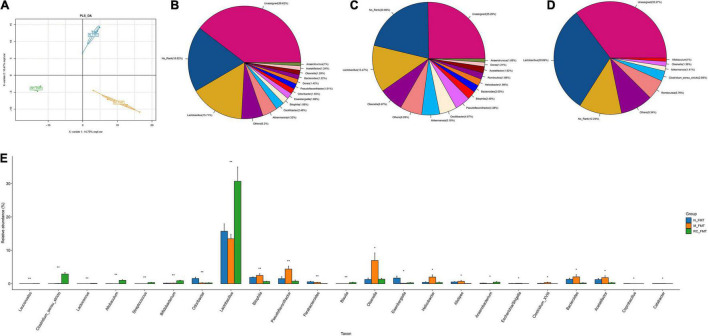
The effects of FMT on GM diversity and abundance in DM mice. **(A)** The differences in community composition studied using PLS-DA. **(B–D)** Pieplot of relative GM abundance at the genus level. **(E)** A histogram showing GM abundance at the genus level. Data are expressed as mean ± SD. *P < 0.05; **P < 0.01; *n* = 5.

Principal Component Analysis (PCA) revealed a clear distinction in gut microbial ecosystem across the N-FMT, M-FMT, and RC-FMT groups, and the altered GM in M-FMT was restored by RC-FMT, close to that in the N-FMT mice (Figure 6A). A total of 23 genera were found to be significantly differentially abundant across the three groups, among which RC-FMT treatment increased the abundance of butyric acid-producing *Clostridium_sensu_stricto* and *Lactobacillus* genera, decreased the amount of *Helicobacter* genera (Figures 6B–E).

### The Effects of *Radix Rehmanniae* and *Cornus Officinalis* Treatment on the Short Chain Fatty Acids/Glucagon-Like Peptide-1 Axis

**FIGURE 7:**
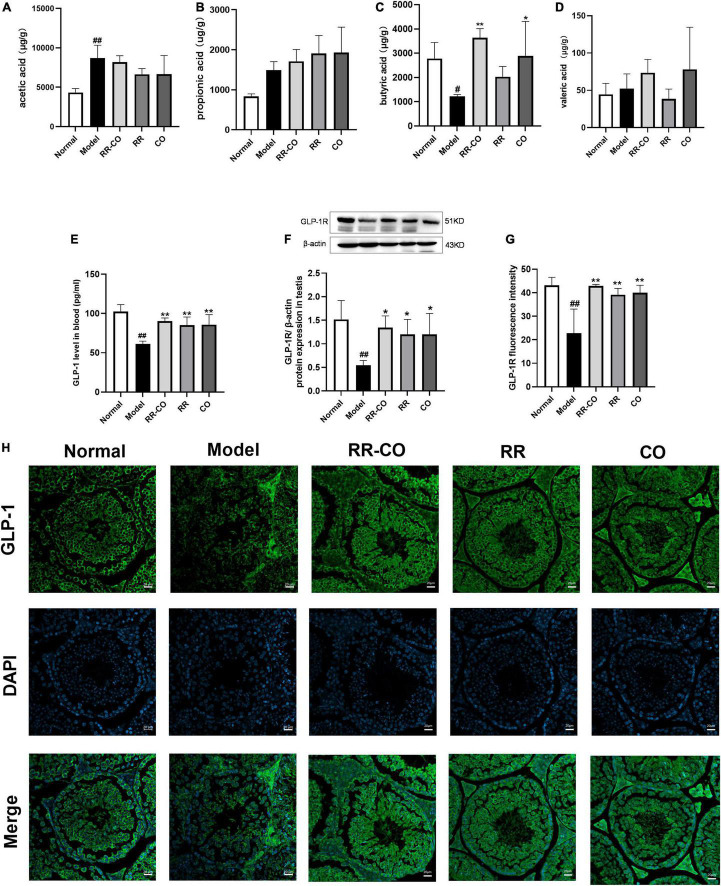
The effects of RR-CO treatment on the SCFAs/GLP-1 axis. **(A–D)** Acetic acid, butyric acid, propionic acid, and isovaleric acid levels in feces (*n* = 5). **(E)** GLP-1 level in blood measured using ELISA (*n* = 4). **(F)** The expression of GLP-1R in testis measured using western blotting (*n* = 3). **(G,H)** Fluorescence intensity of GLP-1R in testis (*n* = 3). Scale bar, 20 μm. Data are expressed as mean ± SD. ^#^*P* < 0.05; ^##^*P* < 0.01 vs. normal group. *P < 0.05; **P < 0.01 vs. model group.

Several studies have reported that the occurrence and development of T2DM are closely related to changes in GM composition and function. GM produces SCFAs, such as acetic acid, propionic acid, and butyric acid. SCFAs promote the secretion of Glucagon-like peptide-1 (GLP-1) by the intestinal L-cells (Wang et al., 2020). Therefore, we evaluated the effect of 8 weeks of administration of RR-CO on the SCFA/GLP-1 axis. The results of GS-MS demonstrated that the content of butyric acid in colon feces in the model group significantly decreased compared to that in the normal group; nevertheless, RR-CO and CO significantly increased the content of butyric acid in the model group. There were no significant differences between the model group and the RR-CO, RR, and CO groups in the levels of acetic acid, propionic acid, and isovaleric acid (Figures 7A–D). To further explore the relationship between GM-mediated regulation of SCFAs/GLP-1 axis and testicular inflammation in diabetes, we detected GLP-1 levels in serum and GLP-1R expression in testicular tissue. Compared to the normal group, the serum GLP-1 level and GLP-1R expression in the model group markedly diminished. Reciprocally, administration of RR-CO, RR, and CO for 8 weeks up-regulated the levels of GLP-1 and GLP-1R expression compared to the model group (Figures 7E–H).

### Correlation of Gut Microbiota With Fasting Blood Glucose, Sperm Damage Indicators, Butyric Acid, and Glucagon-Like Peptide

**FIGURE 8:**
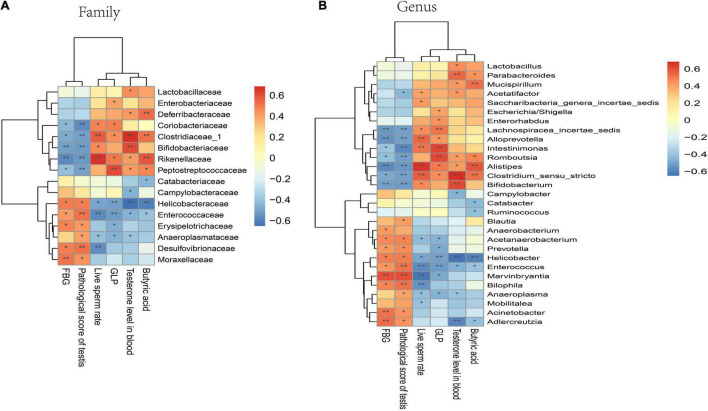
Correlation of GM with FBG, sperm damage indicators, butyric acid, and GLP in mice. **(A)** Correlation analysis at the family level. **(B)** Correlation analysis at the genus level. Significant correlations were noted using adjusted. *P < 0.05; **P < 0.01.

A correlation heatmap was used to assess the association between GM and FBG, sperm damage indicators, butyric acid, and GLP. Based on the heatmap, at the family level, the abundance of *Moraxellaceae*, *Desulfovibrionaceae*, *Erysipelotrichaceae*, *Enterococcaceae*, and *Helicobacteraceae* showed a positive correlation with FBG and pathological testicular score, while *Clostridiaceae_1*, *Coriobacteriaceae, Bifidobacteriaceae*, *Rikenellaceae*, and *Peptostreptococcaceae* abundance exhibited a negative correlation with FBG and pathological testicular score. However, *Rikenellaceae*, *Clostridiaceae_1*, and *Bifidobacteriaceae* abundance demonstrated a positive correlation with live sperm rate and testosterone level in the blood. The abundance of *Rikenellaceae*, *Clostridiaceae_1*, and *Deferribacteraceae* demonstrated a positive correlation with butyric acid and GLP content (Figure 8A). At the genus level, butyric acid producing related bacteria *Clostridium_sensu_stricto* exhibited significantly positive correlation with live sperm rate, testosterone level, butyric acid and GLP level, but a negative correlation with FBG and pathological testicular score. Some conditionally pathogenic bacteria such as *Helicobacter*, *Enterococcus*, and *Marvinbryantia* were positively correlated with FBG and pathological testicular score, but negatively correlated with live sperm rate and testosterone level (Figure 8B).

### Sodium Butyrate Directly Attenuated Testicular Damage and Inflammatory Responses in Diabetes Mellitus Mice

**FIGURE 9:**
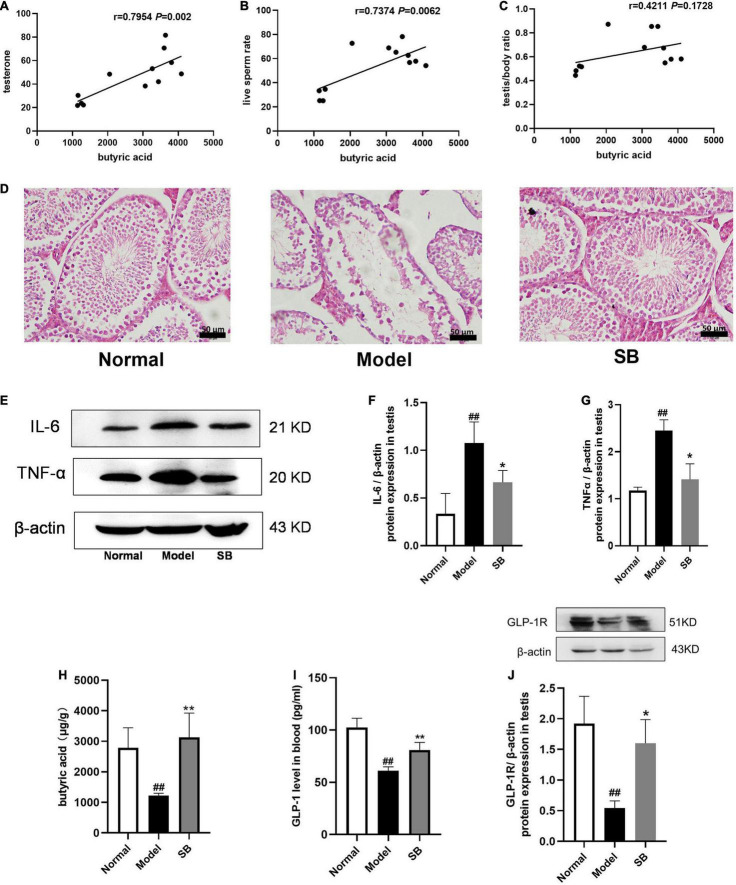
The effects of sodium butyrate on testicular damage and inflammatory responses in DM mice. **(A–C)** Correlation of butyric acid with sperm damage indicators. **(D)** H&E staining of mice testicular tissues (*n* = 4). Scale bar, 50 μm. **(E–G)** The expression of IL-6 and TNF-α in testicular tissue of DM mice (*n* = 3). **(H)** The level of butyric acid in feces (*n* = 5). **(I)** GLP-1 level in blood (*n* = 4). **(J)** The expression of GLP-1R in testis (*n* = 3). Data are expressed as mean ± SD. ^##^*P* < 0.01 vs. normal group. *P < 0.05; **P < 0.01 vs. model group.

Since RR-CO significantly increased the content of butyric acid in feces of diabetic mice, we conducted correlation analysis of butyric acid and sperm damage parameters, and the results revealed that the content of butyric acid in feces was positively correlated with testosterone, live sperm rate, and testis/body weight ratio (Figures 9A–C). To further confirm the role of butyric acid in alleviating reproductive damage in diabetes, sodium butyrate (SB) was intraperitoneally administered to diabetic mice to observe its alleviating effects on diabetic symptoms, testicular damage, and inflammation. After 8 weeks of administration, SB significantly improved the pathological damage to the testis and reduced the expression of inflammatory factors IL-6 and TNF-α in the model group (Figures 9D–G). In addition, compared to the model group, the content of butyric acid in colon feces and GLP-1 in the blood of the mice in the SB group increased significantly. Also, SB significantly up-regulated the expression of GLP-1R in the testicular tissue (Figures 9H–J).

## Discussion

*Radix Rehmanniae* and *Cornus Officinalis* is commonly used in Shen-qi-wan and Zi-cui-yin preparations and is useful in nourishing the yin and tonifying the kidney (Chen et al., 2017). The main physiological function of the kidney is closely related to reproduction in TCM. A growing body of evidence suggested that the RR-CO combination helps in the prevention and treatment of renal disease by improving the renal pathological morphology and ameliorating the damage to renal podocytes, endothelial cells, and mesangial cells (Lv et al., 2016). Thus, in the current study, the protective effects of RR-CO on reproductive injury in DM were explored to validate the TCM theory of the “kidney governing reproduction.” KK-Ay mice, which were generated by transferring a yellow obesity gene (Ay allele) into KK/Ta mice, were selected to spontaneously exhibit a T2DM phenotype associated with hyperglycemia, glucose intolerance, and insulin resistance (Nishimura, 1969). In addition, KK-Ay mice fed with a high-fat diet showed pathological damage to the male reproductive system with morphological and functional impairment of the reproductive system in this study. As expected, we observed that RR-CO treatment improved the pathological and ultrastructural damage to the testis induced in DM by attenuating the structural disorder of spermatogenic tubules and the destruction of tight junctions between cells. Besides, RR-CO, RR, and CO treatments also reduced the FBG level and increased the live sperm rate, serum and testicular testosterone level, serum FSH level and testis/body weight ratio to varying degrees. The therapeutic effects of RR-CO were better than that of RR and CO individually. These data indicated that the herb pair RR-CO possesses a protective effect and alleviates testicular damage caused by DM, but the underlying mechanism needs to be further studied.

Increasing evidence suggests that GM is associated with several chronic metabolic diseases, including obesity (Gasmi et al., 2021), T2DM (Bordalo et al., 2017), hypertension (Verhaar et al., 2020), and non-alcoholic fatty liver disease (Safari and Gerard, 2019). With the increase in clinical studies, the relationship between GM and T2DM has become more and more clear. The diversity and abundance of GM in T2DM patients are known to decrease significantly (Adeshirlarijaney and Gewirtz, 2020). The abundance of *Bifidobacterium*, *Bacteroides*, *Faecalibacterium*, *Akkermansia*, and *Roseburia* negatively correlated with T2DM, while that of *Ruminococcus, Fusobacterium*, and *Blautia* positively correlated with T2DM (Gurung et al., 2020). The abundance of *Bacteroidetes* and *Firmicutes* in DM models varied significantly across studies. Zhu et al. (2020) reported that *Bacteroidetes* and *Alistipes* in the intestine of T2DM rats were down-regulated, while *Firmicutes* and *Blautia* were up-regulated, and *Firmicutes/Bacteroidetes* ratio significantly increased. Another study (Gurung et al., 2020) showed the opposite results – the abundance of *Firmicutes* decreased and that of *Bacteroidetes* increased in the T2DM group. In this study, the relative abundance of *Firmicutes* and *Bacteroidetes* did not show significant differences between the normal, model, and RR-CO group. The inconsistency in the results may be due to the interactions across the different gut microbes.

In the in-depth studies of the interactions between GM and the host, the GM has been found to interact not only locally with the gut but also with several distal organs and tissues. A series of hypotheses emphasized the pivotal role of the GM in the gut-brain axis (Morais et al., 2021), gut-liver axis (Albillos et al., 2020), gut-muscle axis (Liao et al., 2020), and gut-renal axis (Coppo, 2018). Recently, the gut-testis axis has received substantial attention, which closely associates the testicular function to the regulation of GM *via* host metabolites. Several studies have shown that androgens significantly reshape the GM through complex pathways (Harada et al., 2016; Zhou et al., 2020). In turn, the GM has been found to regulate androgen production and metabolism (Collden et al., 2019). Several FMT studies have shown that GM regulates sperm production and reproductive function. Ding et al. (2020) reported that FMT from high-fat diet-fed mice disrupted spermatogenesis and reduced sperm motility. The mechanism may be related to *Prevotella copri*-mediated endotoxin release induced by a high-fat diet (Ding et al., 2020). Another study showed that transplanted feces from alginate oligosaccharide-fed mice to busulfan-treated mice restored the jeopardized spermatogenic function (Zhang et al., 2021a). In the present study, testicular pathomorphological damage was aggravated after fecal transplantation to pseudo-sterile mice in the diabetic model group, showing disorganized spermatogenic cell structure and a reduced number of mature spermatogenic cells in the lumen, while mice in the RC-FMT group showed significantly reduced testicular pathological damage and increased live sperm rate. Likewise, feces from the RC-FMT group decreased the abundance of *Helicobacter genera* in the M-FMT group. Furthermore, RC-FMT increased the abundance of the probiotics *Lactobacillus genera* and butyric acid-producing *Clostridiaceae_1* genera. Hence, RR-CO may be beneficial to target gut microbial regulation to treat DM-induced reproductive damage, including male sexual dysfunction, hypogonadism, and even infertility.

Most TCMs are in the oral dosage form, and GM plays an important role in the efficacy of these drugs. A growing body of evidence indicates that TCMs interact with GM after oral administration, causing alterations in the composition and metabolites produced by GM. Zhang et al. (2021c) demonstrated that the RR-CO combination remarkably increased the abundance and diversity of GM in rats with adenosine-induced chronic kidney disease. CO extract selectively modulated the structure of GM, thereby alleviating T2DM (Niu et al., 2020). Qin-xin-tang increased the SCFA-producing flora in the gut of T2DM rats, thus, up-regulating the secretion of SCFA, increasing intestinal integrity, inhibiting inflammatory response, and improving insulin resistance (Wei et al., 2018). In the current study, the Shannon index was elevated whereas the Simpson’s index was decreased in the RR-CO group compared to the model group, illustrating that RR-CO treatment increased GM diversity in DM mice. In addition, according to the PLS-DA results, there were significant structural differences in GM across the normal, model, and RR-CO groups. Further study of the differences in GM structure across the groups revealed that the relative abundance of *Clostridiaceae_1* increased and that of *Catabacteriaceae* and *Helicobacteraceae* decreased in the RR-CO at the family level, RR, and CO groups. The therapeutic effect of RR-CO was superior to that of the individual herbs. Further heatmap analysis showed that the abundance of *Clostridiaceae_1* was negatively correlated with FBG and pathological testis score and positively correlated with live sperm rate and testosterone level. The abundance of *Helicobacteraceae* was positively correlated with FBG and pathological testis score. Moreover, at the genus level, RR-CO restored the increased abundance of *Catabacter, Marvinbryantia*, and *Helicobacter* in the model group. Also, RR-CO treatment reversed the reduction in the abundance of *Clostridium_sensu_stricto* in the model group. All these results suggested that RR-CO may alleviate the diabetic reproductive damage by modulating the structure of GM, increasing the abundance of beneficial GM, and decreasing that of pathogenic GM.

Recent studies have revealed that GM is closely related to the occurrence of T2DM through SCFA metabolism, bile acid metabolism, branched-chain amino acid metabolism, lipopolysaccharide secretion, etc. (Forslund et al., 2017; Salazar et al., 2020; Zheng et al., 2021). In particularly, SCFAs, including acetic acid, propionic acid, and butyric acid, are metabolites of fermentation of dietary fiber produced by the intestinal flora. Wang et al. (2017) showed that modulation of GM contributed to the production of SCFA, particularly butyric acid, which inhibits the secretion of pro-inflammatory factors, such as IL-6 and TNF-α. Besides, the binding of SCFAs to G protein-coupled receptor (GPR) 41 and GPR43 induces intestinal secretion of GLP-1, attenuating inflammatory response and protecting islet β-cells (Veprik et al., 2016). In the current study, we found that IL-6 and TNF-α expression increased significantly in testicular and colonic tissues in the model group, and RR-CO down-regulated their expression. Simultaneously, GS-MS results showed that RR-CO dramatically increased butyric acid level in the colonic feces in the model group, whereas it had no significant effect on acetic acid, propionic acid, and isovaleric acid levels. Similarly, the heatmap results also indicated that butyric acid-producing bacterium *Clostridiaceae_1* abundance increased by RR-CO was positively correlated with butyric acid levels. GLP-1 has been reported to play an important function in the treatment of T2DM and its complications, such as regulating glucose homeostasis, gastrointestinal motility, and food intake. GLP-1 releases from gut enteroendocrine cells and exerts its effects through the activation of the G protein-coupled receptor GLP-1R and its secretion is facilitated by butyric acid (Graaf et al., 2016). Thus, we further measured the levels of GLP-1 in the serum and GLP-1R in the testicular tissue and discovered that GLP-1 and GLP-1R expression was reduced in the model group and their expression was restored by RR-CO. To further investigate the role and mechanism of the metabolite butyric acid in alleviating diabetic reproductive damage, SB was administered intraperitoneally to diabetic mice. The results demonstrated that SB greatly decreased the expression of IL-6 and TNF-α in the testes of diabetic mice and attenuated testicular pathological damage. Along with the increase in butyric acid level in the feces, the expression of GLP-1 in serum and GLP-1R in testis increased in the SB group. All these results suggested that butyric acid reduces testicular inflammation and alleviates testicular pathological damage by regulating intestinal GLP-1 secretion through a mechanism related to the down-regulation of testicular GLP-1R.

## Conclusion

In general, the results from the current study suggested that the herb pair RR-CO improves T2DM-induced reproductive complications, such as lowering FBG, improving pathological damage to the spermatogenic tubules, alleviating the inflammation in the testis, and increasing the live sperm rate, by modulating the composition and function of the GM. The ameliorating effect of RR-CO on testicular inflammation may be associated with the regulation of butyric acid/GLP-1/GLP-1R pathway mediated by GM (Graphical abstract).

## Data Availability Statement

Publicly available datasets were analyzed in this study. This data can be found here: https://doi.org/10.6084/m9.figshare.16929406.v2.

## Ethics Statement

The animal study was reviewed and approved by the Animal Ethics Committee of the Nanjing University of Traditional Chinese Medicine (code: 201903A019).

## Author Contributions

HX, JS, QW, and YC participated in research design. YC, LL, MJ, and SS conducted the experiments. JJ contributed to new reagents or analytic tools. QW and AS performed the data analysis. YC and HX wrote or contributed to the writing of the manuscript. All authors contributed to the article and approved the submitted version.

## Conflict of Interest

The authors declare that the research was conducted in the absence of any commercial or financial relationships that could be construed as a potential conflict of interest.

## Publisher’s Note

All claims expressed in this article are solely those of the authors and do not necessarily represent those of their affiliated organizations, or those of the publisher, the editors and the reviewers. Any product that may be evaluated in this article, or claim that may be made by its manufacturer, is not guaranteed or endorsed by the publisher.
